# Tuberculosis Masquerading as Behcet's Disease‐Pseudo Bechet's Syndrome: A Case‐Based Review of Literature

**DOI:** 10.1002/rcr2.70469

**Published:** 2026-01-22

**Authors:** Rinoosha Rachel, Naveen Polavarapu, Jithin Mathew, Virender Pratibh Prasad, Venkata Nagarjuna Maturu

**Affiliations:** ^1^ Department of Pulmonology Yashoda Super Speciality Hospitals Hitech city Hyderabad India; ^2^ Department of Gastroenterology Yashoda Super Speciality Hospitals Hitech city Hyderabad India; ^3^ Department of Rheumatology Yashoda Super Speciality Hospitals Hitech city Hyderabad India

**Keywords:** anti‐tubercular therapy, Behcet's disease, Pseudo Behcet's syndrome, tuberculosis

## Abstract

Behcet's disease is a chronic, multisystem variable vessel vasculitis characterised by recurrent oral and genital ulcers, ocular inflammation and a wide range of systemic manifestations. Pseudo‐Behcet's syndrome refers to a condition that mimics these clinical features but arises from distinct etiologies. We present a case of a 33‐year‐old male with year‐long recurrent oral ulcers and intermittent abdominal pain, followed by scrotal ulcers, severe fatigue, weight loss and appetite loss. Chest imaging demonstrated bilateral consolidations with cavitation and right‐sided pleural effusion, prompting consideration of a Behcet's mimic. Thoracoscopic pleural biopsy revealed acid‐fast bacilli, confirming tuberculosis. A diagnosis of Pseudo‐Behcet's secondary to tuberculosis was made. Initiation of anti‐tubercular therapy led to complete resolution of symptoms within 4 months. A literature review identified nine cases (including the index case) in which tuberculosis presented with Behcet's‐like features, underscoring the complex association between Behcet's disease and tuberculosis. This case underscores the need to consider tuberculosis as a Behcet's disease mimic, particularly in endemic areas, to prevent misdiagnosis and inappropriate immunosuppression.

## Graphical abstract

We report a case of recurrent oral/scrotal ulcers with systemic symptoms in a 33‐year‐old man masked pulmonary tuberculosis: imaging showed cavitary consolidations with effusion, and thoracoscopic pleural biopsy demonstrated acid‐fast bacilli. Anti‐tubercular therapy led to complete resolution, underscoring TB as a key Behçet's mimic to exclude before immunosuppression in endemic regions.

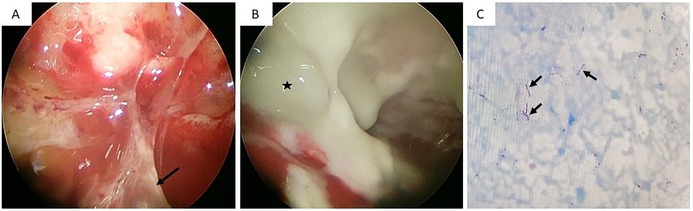

## 1 Introduction

Behcet's disease is a chronic, multisystem inflammatory disorder of unknown aetiology, classified as both systemic vasculitis and a neutrophilic dermatosis, affecting blood vessels of all types and sizes. Clinically, it is characterised by recurrent oral aphthous ulcers, genital ulcers, cutaneous lesions and uveitis [1]. However, pulmonary consolidations/cavities and pleural effusions are distinctly unusual and must direct further evaluation of an alternative aetiology. Pseudo‐Behcet's syndrome is a rare clinical entity that clinically mimics the classic Behcet's disease but is associated with underlying causes mainly infections, neoplasms or drugs [2]. Among the various documented causes of pseudo‐Behcet's syndrome, tuberculosis as an underlying aetiology is rare, with only a limited number of cases reported in the literature to date. It is prudent to distinguish classic Behcet's disease from pseudo‐Behcet's disease, as the treatments for the conditions differ. While Behcet's disease needs immunosuppressants, pseudo‐Behcet's caused by tuberculosis responds well to anti‐tubercular therapy. Using immunosuppression in such cases may worsen the infection, making early and accurate diagnosis essential. This article describes a case of pseudo Behcet's syndrome secondary to tuberculosis and also provides a review of earlier published case reports outlining the clinical characteristics.

## 2 Case Report

A male patient in his early 30s, resident of Somalia, presented to us with complaints of recurrent painful oral ulcerations and intermittent abdominal pain for the last 1 year. Approximately 1 month before presentation, he began experiencing multiple painful scrotal ulcers, accompanied by a pronounced decline in appetite, significant weight loss and debilitating fatigue. He did not have any fever, cough, shortness of breath, joint pains or swelling or redness of the eyes. He did not have any significant past medical history.

**FIGURE 1:**
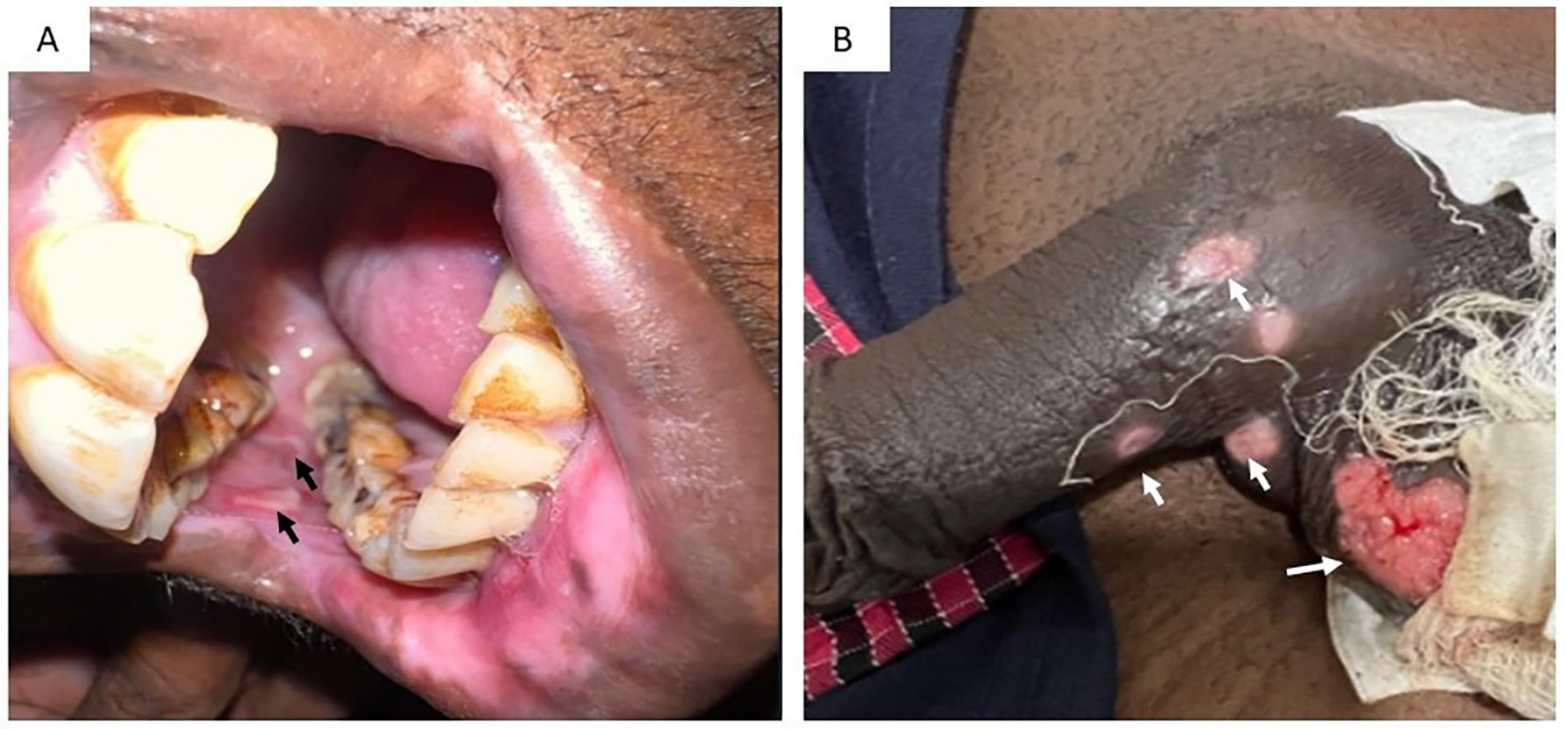
(A) Oral examination showing multiple well‐defined aphthous ulcers (black arrows) over the bilateral buccal mucosa; (B) Genital examination showing a large solitary ulcer over the scrotal skin with erythematous margins (white arrow), and healing ulcers at the penile shaft base (white arrows).

**FIGURE 2:**
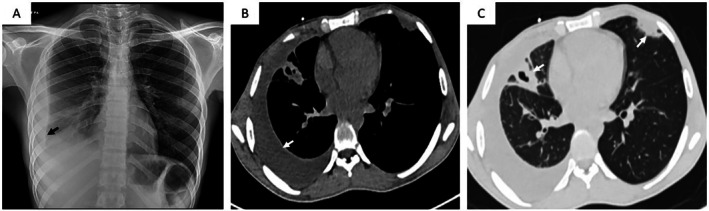
(A) Chest x‐ ray showing right sided effusion with right lower zone haziness (black arrow); (B, C) Mediastinal and lung parenchymal windows showing bilateral lung consolidations and cavities (white arrows) with right sided pleural effusion (black arrow).

At presentation, he was afebrile; tachycardic with pulse rate 115 beats per minute; blood pressure 110/70 mmHg; room air saturation 98% and respiratory rate 16 per minute. He was pale, but there was no icterus, lymphadenopathy, clubbing or cyanosis. Oral cavity examination revealed multiple aphthous ulcers and some deeper ulcerative lesions on the buccal mucosa (Figure 1A). Genitalia examination revealed multiple ulcers over the scrotum (Figure 1B). Respiratory system examination revealed reduced air entry in the right basal area; other systemic examinations were unremarkable. Laboratory investigations showed severe microcytic, hypochromic anaemia (haemoglobin level of 5 g/dL; normal 12–16 g/dL), with a normal total leukocyte count, an elevated platelet count of 630,000/cu mm (normal 150,000–450,000/cu mm), and an elevated C‐reactive protein of 201 mg/L (normal < 5 mg/L). His liver function tests and renal function tests were within normal limits. Chest radiograph showed right‐sided loculated pleural effusion with lower zone haziness (Figure 2A). He underwent a contrast‐enhanced chest computed tomography (CT) scan which revealed bilateral pulmonary consolidations with cavitations, along with a right‐sided pleural effusion (Figure 2B,C).

He underwent upper gastrointestinal endoscopy and colonoscopy for evaluation of anaemia, which showed diffuse ulcerations along with bleeding areas involving the oral cavity, oesophagus, duodenum and pyloric region. Multiple biopsies were obtained during the procedure. Histopathologic examination showed fibrin deposition with neutrophilic exudate and granulation tissue formation, but there was no evidence of viral cytopathic changes, atypical cells or vasculitis. Infectious workup, including tests for HIV, hepatitis B and C, syphilis and herpes simplex virus, was negative. Given the mucocutaneous manifestations, there was an initial high clinical suspicion for Behcet's disease; other autoimmune markers including ANA by immunofluorescence, immunoblot and HLA‐B51 were negative. Pathergy test was performed and was negative.

Chest ultrasound revealed a moderate right‐sided pleural effusion with multiple septations. Diagnostic thoracocentesis yielded serosanguinous pleural fluid with a protein level of 5.2 g/dL (normal < 3.0 g/dL), adenosine deaminase (ADA) of 37 U/L (normal < 40 U/L), a total cell count of 200 cells/mm^3^ (normal < 1000 cells/mm^3^) with 80% polymorphonuclear cells (normal < 25%) and 20% lymphocytes (normal < 75%) and a glucose level of 79 mg/dL (normal > 60 mg/dL and approximately equal to serum glucose). Microbiological investigations, including Xpert MTB/RIF, acid‐fast bacilli smear, bacterial culture and fungal smear, were all negative.

**FIGURE 3:**
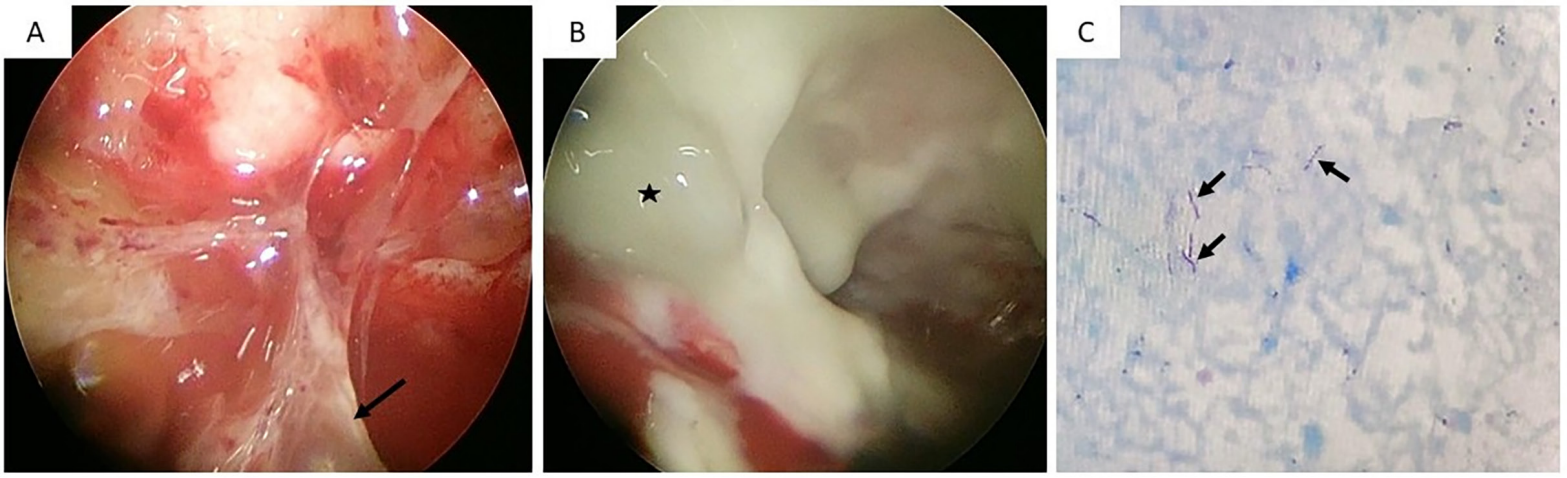
(A, B) Medical thoracoscopy view of the pleural cavity revealing multiple fibrous septations (black arrow), dense pleural adhesions and localised pockets of purulent fluid (star symbol) within the pleural cavity; (C) Ziehl‐Neelsen staining of the pleural tissue demonstrating the presence of acid‐fast bacilli (black arrow).

He subsequently underwent medical thoracoscopy under local anaesthesia, which revealed multiple septations, dense pleural adhesions and localised pockets of pus within the pleural cavity (Figure 3A,B). Microbiological examination of pleural tissue was positive for Xpert MTB and Acid‐fast bacilli (AFB) smear. Histopathological examination of the pleural tissue revealed necrotising granulomatous inflammation with lympho‐histiocytic infiltrates, and acid‐fast bacilli on Ziehl‐Nelsen staining, suggesting a tubercular pleural effusion (Figure 3C).

The integration of these clinical, radiological, endoscopic and histopathological findings led to the diagnosis of pseudo‐Behcet's syndrome—a rare condition that mimics Behcet's disease and a complication of tuberculosis infection. He underwent a comprehensive ophthalmic examination to look for evidence of ocular involvement. There was no evidence of uveitis or retinal vasculitis.

He was started on all oral anti‐tubercular therapy with rifampicin (600 mg/day), isoniazid (300 mg/day), ethambutol (1000 mg/day) and pyrazinamide (1500 mg/day). Haemoglobin was stabilised with multiple blood transfusions. At the time of discharge, the patient was clinically stable and was advised to continue regular anti‐tubercular therapy. Within 2 weeks of initiating treatment, existing ulcerative lesions began to slowly regress, and no new ulcerations developed. He returned to his home country after a month of therapy. He is regularly being followed up over the phone to ensure drug compliance and resolution of symptoms. At 4 months follow up, there was complete resolution of symptoms, resolution of genital and oral ulcers, and stabilisation of haemoglobin levels. There is no recurrence so far, and he is planned to complete the 6 months of ATT.

This case emphasises the importance of a comprehensive evaluation in patients presenting with multisystem ulcerative lesions and the need to consider infectious causes in the differential diagnosis of Behcet's‐like syndromes.

A comprehensive search of the PubMed, EmBase and google scholar databases using the keywords “pseudo‐Behçet's syndrome” OR “Behcet's syndrome” OR “Bechet's disease” and “pulmonary tuberculosis” OR “tuberculosis” identified eight case reports that specifically detail clinical presentations of pseudo‐Behçet's syndrome secondary to tuberculosis infection. A summary of published cases of pseudo‐Behcet's syndrome secondary to *Mycobacterium tuberculosis* is provided in Table 1 [3, 4, 5, 6, 7, 8, 9]. Among the reviewed cases, patients ranged in age from early 20s to late 40s, with a nearly equal male‐to‐female distribution. The most common clinical manifestations included oral and genital ulcers, erythema nodosum‐like skin lesions, arthralgia or arthritis, lymphadenopathy and constitutional symptoms like fever, weight loss. Interestingly, ocular involvement which is considered an important diagnostic feature of classical Behcet's disease, was largely absent in the reviewed cases, with the exception of a single report by Zhang et al. [7], where anterior uveitis was documented.

**TABLE 1:** Clinico‐radiologic features and outcomes of published cases of tuberculosis associated pseudo Behcet's disease.

Author (year)	Age/sex demography	Clinical presentation	Duration of symptoms	Diagnosis confirmation	Associated tuberculosis	Management, outcome and follow up*
Hamill et al. (2006) [4]	25F, Japan	Oral ulcers, genital ulcers, acne, low back pain, cough, pleuritic chest pain	5 months	Sputum smear—positive for MTB	Pulmonary tuberculosis	Standard ATT, complete resolution, no relapse after 36 months of follow up
Hamill et al. (2006) [4]	21F, India (resident of UK)	Oral ulcers, genital ulcer, EN like nodules, fever, arthralgia, pleuritic chest pain, axillary lymphadenitis	3 years	HRCT Chest‐ Miliary Tuberculosis Bone marrow aspiration‐caseating granuloma FNAC Lymph node‐ AFB smear Positive	Disseminated tuberculosis	Standard ATT, Complete resolution, no relapse after 24 months of follow up.
Sharma et al. (2013) [5]	45 M, India	Oral and genital ulcers, polyarthritis, EN like nodules on lower limbs, fever, pseudofolliculitis.	4 years	Skin biopsy of nodules on legs—S/O papulo necrotic tuberculids (AFB negative, PCR positive for gene IS6110) Mantoux test‐strong positive	Extra Pulmonary tuberculosis (Poncet's disease)	Standard ATT, Complete resolution, no relapse after 9 months of follow up
Shinoda et al. (2014) [3]	45F, Japan	Oral/genital ulcers, EN‐like lesions, migratory arthralgia, fever, cervical lymph nodes	3 years	Mantoux test‐positive Lymph node biopsy‐granulomas with caseous necrosis. Skin biopsy‐papulonecrotic tuberculids	Tuberculous lymphadenitis	Standard ATT, Complete resolution, no relapse after 40 months of follow up.
Fukui et al. (2014) [6]	48 M, Japan	Oral and genital ulcers, EN like nodules, multiple lymph nodes in neck.	10 years	Lymph node biopsy—Caseous epithelioid granulomas, Positive for AFB in stain and culture	Tuberculous lymphadenitis	Standard ATT, Complete symptom resolution, follow up duration‐ NA
Zhang et al. (2015) [7]	45F, China	Genital ulcer, uveitis, arthritis, EN‐like rash. Past history of pleural tuberculosis	3 years	Lymph node biopsy‐caseating granulomas, AFB positive	Tuberculous lymphadenitis	Standard ATT, complete resolution and no recurrence after 12 months of follow up
Lim et al. (2022) [8]	26 M, Bangladesh	Oral/genital ulcers, indurated skin nodules, fever	1 month	HRCT chest – Tree in bud appearance in left lower lobe Sputum—AFB Smear negative, Xpert MTB positive, AFB culture positive	Pulmonary tuberculosis	Standard ATT, Complete symptom resolution, follow up duration‐ NA
Choudhary et al. (2023) [9]	20sM, India	Penile ulcer, oral ulcers, pustular eruptions on skin, inguinal and cervical lymph nodes	5 months	Mantoux test—strong positive Skin biopsy‐inconclusive. Lymph node biopsy—inconclusive HRCT chest—left upper lobe consolidation BAL‐ AFB stain positive	Pulmonary tuberculosis	Standard ATT, Complete healing of lesions, under follow up.
Index case	33 M, Somalia	Oral and penile ulcers, gastric ulcers, anorexia, weight loss abdominal pain	1 year	HRCT chest‐ right lower lobe consolidation and effusion. Pleural biopsy—granulomatous inflammation, AFB stain positive.	Pleuropulmonary tuberculosis	Standard ATT, Near complete resolution of symptoms, under follow up

Abbreviations: AFB: acid fast bacillus; ATT: anti‐tuberculosis therapy; BAL: Broncho alveolar lavage; EN: erythema nodosum; F: female; FNAC: fine needle aspiration cytology; HRCT: high resolution computed tomography; M: male; MTB: *Mycobacterium tuberculosis* ; PCR: polymerase chain reaction.

* Standard ATT: Intensive phase of 4 drugs for 2 months followed by maintenance phase of 2/3 drugs for 4 months.

An important epidemiological insight is that the majority of reported cases of Pseudo‐Behcet's syndrome secondary to tuberculosis are from tuberculous‐endemic regions, particularly across the Asian and African continents. This highlights the need for heightened clinical vigilance when evaluating Behcet's‐like presentations in such settings.

Both pulmonary and extrapulmonary disease presentations were seen. While pulmonary involvement was frequently observed, lymph node tuberculosis was the most common extrapulmonary manifestation [3, 6, 7]. Among the reviewed cases, tuberculosis was confirmed either through histopathology [3, 6, 7], molecular evidence (PCR based detection of MTB DNA) [5, 6] or microbiological tests (AFB stain or AFB culture) [4, 8, 9]. These findings underscore the importance of tissue sampling and microbiologic confirmation in patients with atypical Behcet's‐like symptoms, particularly when there are marked constitutional symptoms or pleuro‐pulmonary involvement.

In most published cases, prolonged diagnostic delays of up to several years were reported, highlighting the need for early consideration of tuberculosis in the differential diagnosis of Behcet's‐like syndromes in appropriate settings. All patients received standard anti‐tubercular therapy (typically 2 months of intensive therapy followed by 4–6 months of continuation therapy). All the cases showed complete resolution of symptoms without recurrence on follow‐up, supporting the infectious cause rather than autoimmune nature as the cause of their presentations.

## 3 Discussion

Pseudo‐Behcet's syndrome is a rare clinical entity that clinically mimics the classic Behcet's disease but is associated with underlying infections, neoplasms or drugs [2]. Among the various documented causes of pseudo‐Behcet's syndrome, tuberculosis as an underlying aetiology is rare, with only a limited number of cases reported in the literature to date. The pathogenesis of tuberculosis associated pseudo‐Behcet's disease is unclear. One potential mechanism involves molecular mimicry, a process where the immune system confuses self‐antigens with microbial antigens, leading to an autoimmune response. In primary Behcet's disease, human 60‐kD heat shock protein (HSP60) has been identified as a candidate autoantigen which is overexpressed in active lesions such as oral ulcers, erythema nodosum‐like skin lesions and intestinal ulcers [1, 3, 10]. There is a high degree of homology between human heat shock protein 60 (HSP60) and the *Mycobacterium tuberculosis* ‐derived HSP65 antigen [10]. This molecular similarity may trigger immunological cross‐reaction, wherein an immune response directed initially against the microbial HSP65 inadvertently targets the host's own HSP60 [3]. Such molecular mimicry is believed to play a pivotal role in the development of Behcet's disease‐like syndromes, particularly in individuals with genetically predisposed immune background [3, 10].

Differentiating pseudo‐Behcet's syndrome from classic Behcet's disease is essential because their management differs markedly; while Behcet's disease is managed with immunosuppressive therapy, pseudo‐Behcet's syndrome requires prompt initiation of anti‐tubercular treatment, and the use of immunosuppressants in such cases could exacerbate the underlying infection.

Pulmonary involvement is uncommon in Behcet's disease (less than 1%) and when present usually manifests as pulmonary artery aneurysms, pulmonary thromboembolism or pulmonary infarcts [11]. Pleural effusions, consolidations, cavities, miliary and centrilobular nodules are uncommon in Behcet's and when present should alert the clinician. Significant constitutional symptoms, cough with sputum production and cervical lymphadenopathy are uncommon in Behcet's and when present should raise the suspicion of underlying tuberculosis, especially when the index case hails from a country endemic for tuberculosis. Pathergy test (a non‐specific hyperreactivity) is considered a characteristic feature of true Behcet's disease and is typically absent in pseudo‐Behcet's disease. Residence in a country endemic for tuberculosis, negative pathergy test, negative autoimmune work up and presence of pulmonary cavities, pleural effusions or cervical lymphadenopathy are pointers towards tuberculosis as a cause of pseudo‐Bechet's syndrome in patients presenting with recurrent oro‐genital ulcers.

The association between Behcet's disease and tuberculosis is complex and multi‐dimensional. There are three distinct clinical scenarios: (a) tuberculosis as a complication of immunosuppressive treatment of true Behcet's disease; (b) tuberculosis presenting as Behcet's disease–Pseudo Behcet's syndrome and (c) co‐existence of true Behcet's disease and active clinical tuberculosis. A brief description of the three scenarios is provided here.

- *Tuberculosis complicating Behcet's disease*: Treatment of Behcet's disease requires steroids and other immunosuppressive medicines like tumour necrosis factor alpha inhibitors. Pulmonary or disseminated tuberculosis can occur due to these immunosuppressive medicines (re‐activation of latent tuberculosis or new infection), in tuberculosis endemic countries [12, 13]. It has also been shown that there are higher odds of tuberculosis infection due to immunosuppressive medicines in Behcet's disease when compared to other rheumatologic diseases [14]
- *Tuberculosis presenting as Pseudo‐Behcet's syndrome*: As shown in the index case, the clinical presentation of active tuberculosis can sometimes mimic Behcet's disease, and this is called “Pseudo Behcet's syndrome”. In Pseudo Behcet's syndrome, there is no true Behcet's vasculitis. Therapy with anti‐tuberculous medicines (ATT) alone will lead to complete resolution of symptoms and signs.
- *Co‐existence of true Behcet's disease and active tuberculosis*: It is also likely that these two diseases can co‐exist and be diagnosed simultaneously or sequentially, especially in tuberculous endemic countries of the world [15, 16]. When both diseases co‐exist, treatment would require a combination of immunosuppressive medications and ATT. The co‐existence of these two diseases can be by chance or due to shared genetic predisposition for these two diseases [13].

To summarise, the learning points from the index case are the following: (a) In patients presenting with recurrent oro‐genital ulcers and a Bechet's like presentation, a thorough systemic evaluation needs to be performed to identify clinical findings which may point towards an alternative aetiology; (b) In regions with high tuberculosis endemicity, pseudo‐Behcet's syndrome secondary to tuberculosis should be considered in the differential diagnosis of patients presenting with Behcet's‐like features; (c) Presence of cervical lymphadenopathy, loculated pleural effusions or pulmonary cavities would point towards underlying tuberculosis as a cause of pseudo‐Bechet's disease; (d) Timely initiation of anti‐tubercular therapy can lead to complete resolution of symptoms in pseudo‐Behcet's syndrome secondary to tuberculosis, highlighting the importance of accurate diagnosis to avoid potentially harmful immunosuppression.

## Author Contributions

R.R. performed thoracoscopy, drafted the manuscript. N.P. performed endoscopy, managed the patient. J.M. managed the patient, reviewed manuscript. V.P.P. managed the patient, reviewed manuscript. V.N.M. managed the patient and reviewed the manuscript.

## Consent

The authors declare that written informed consent was obtained for the publication of this manuscript and accompanying images using the consent form provided by the Journal.

## Conflicts of Interest

The authors declare no conflicts of interest.

## Data Availability

The data that support the findings of this study are available from the corresponding author upon reasonable request.
